# Case Report: Hemophagocytic lymphohistiocytosis secondary to *Escherichia coli* infiltration of bone marrow in a patient with seronegative rheumatoid arthritis treated with low-dose methotrexate

**DOI:** 10.3389/fimmu.2026.1863594

**Published:** 2026-07-13

**Authors:** Ziping Liu, Fengjian Li, Yunhong Liu, Xueyan Chen

**Affiliations:** Department of Clinical Laboratory Medicine, The People’s Hospital of Longhua, Shenzhen, Guangdong, China

**Keywords:** *Escherichia coli*, immunosuppression, infiltration of bone marrow, secondary hemophagocytic lymphohistiocytosis, sepsis

## Abstract

Hemophagocytic lymphohistiocytosis (HLH) is a disorder characterized by dysregulated immune activation triggered by various etiologies, leading to excessive inflammatory responses, cytokine storm, and subsequent organ dysfunction. While the presence of hemophagocytic cells in septic shock is generally considered a physiological response that seldom requires specific intervention, the finding of abundant hemophagocytes along with *Escherichia coli*(*E. coli*) on a bone marrow smear warrants careful assessment for possible HLH. We herein present a fatal case of HLH secondary to an E. coli urinary tract infection in a seronegative rheumatoid arthritis patient on low-dose methotrexate. The bone marrow smear not only demonstrated typical hemophagocytes but also revealed phagocytes containing ingested E. coli. In contrast to previously reported cases, *E. coli* in this patient not only caused severe bacteremia but was also demonstrated on the bone marrow aspirate, resulting in profound immunosuppression.

## 1 Introduction

Hemophagocytic lymphohistiocytosis (HLH) is a life-threatening condition increasingly identified in adults, presenting as a severe multisystem disorder that typically occurs in individuals with various underlying diseases (1). HLH is categorized into two principal groups based on specific triggers or underlying etiologies: HLH disease mimics​ and HLH disease (2). The latter can be further subclassified into the following etiologically defined subtypes: malignancy-associated HLH, iatrogenic/immune activation therapy–associated HLH, immune deficiency–associated HLH, and HLH with no specific association (2). The essence is a syndrome of excessive/hyperinflammatory response caused by hereditary or acquired immune regulatory dysfunction (3). Sepsis can progress to infection-associated HLH if the inflammatory response becomes excessively intense, advancing from systemic inflammatory response syndrome (SIRS) to severe hyperinflammation or cytokine storm.

HLH can be challenging to diagnose, as it may resemble septic shock with multiple organ failure (4). When HLH is suspected, performing a bone marrow aspiration, which serves as the gold standard for diagnosis, is highly necessary. The presence of hemophagocytic cells in septic shock is typically a physiological response and generally does not require specific intervention (5). However, when a significant number of hemophagocytes appear in the bone marrow smear, careful evaluation is required to determine whether HLH has occurred.

Here, we report a case of fatal HLH secondary to a urinary tract infection (UTI) caused by *E. coli* in a seronegative rheumatoid arthritis (RA) patient receiving low-dose methotrexate (MTX) therapy. The bone marrow smear not only revealed typical hemophagocytes but also showed phagocytes that had engulfed a number of *E. coli*.

## 2 Case presentation

In November 2025, a 59-year-old woman presented with a four-month history of recurrent joint swelling and restricted mobility. The diagnosis of seronegative rheumatoid arthritis (RA) was established based on elevated inflammatory markers (CRP: 110 mg/L; ESR: 60 mm/h) and imaging evidence of synovial thickening in the right wrist joint with associated triangular fibrocartilage complex(TFCC) injury, despite negative RF and anti-CCP antibody serology. The patient complained of urinary frequency and urgency. While *E. coli* was identified on urine culture, no intervention was pursued as the bacterial colony count was < 1000 CFU/mL. The patient was started on low-dose oral methotrexate (10 mg once weekly) along with prednisone acetate (15 mg twice daily). Notably, folic acid supplementation was not administered concurrently. Laboratory investigations revealed leukocytosis (13.4×10^9^/L) with 86% neutrophils, hemoglobin 134 g/L, platelet count 296×10^9^/L, and normal hepatic and renal function. Approximately four weeks later, she visited the emergency department with progressively worsening abdominal pain and diarrhea of unknown etiology. Her vital signs, including body temperature and blood pressure, were within normal limits. Physical examination on admission revealed a soft abdomen, though tenderness, rebound tenderness, and muscle guarding were present, along with a positive Murphy’s sign. Multiple skin ecchymoses and gingival bleeding were also noted. Abdominal ultrasound showed mild splenomegaly and cholelithiasis. Systemic inflammation was indicated by markedly elevated levels of C−reactive protein, interleukin−6, serum amyloid A, and procalcitonin. A complete blood count revealed severe pancytopenia, with a white blood cell count of 0.7×10^9^/L, an absolute neutrophil count of 0.27×10^9^/L, hemoglobin of 103 g/L, and platelet count of 1×10^9^/L. Peripheral blood smear further confirmed pancytopenia, with no morphologically abnormal cells observed. Laboratory investigations, including assessments of liver and kidney function, triglyceride levels, and serum ferritin, demonstrated significant abnormalities. Urinalysis showed the presence of red blood cells and phagocytes, with no evidence of pyuria. Abnormal laboratory findings are shown in **Table 1**. The patient was initially diagnosed with sepsis, methotrexate-induced pancytopenia, cholecystolithiasis with acute cholecystitis, pneumonia based on thoracic imaging, and acute kidney injury, and subsequently transferred to the Intensive Care Unit (ICU). Antimicrobial therapy with intravenous meropenem (1.5g/day) and vancomycin (2g/day) was initiated empirically. Additionally, the patient received symptomatic therapies, including granulocyte colony-stimulating factor, thrombopoietin, blood component transfusions, and leucovorin rescue. Methotrexate plasma concentration measured 0.1 µmol/L, a level that does not exclude the possibility of drug-induced pancytopenia.

**Table 1:** Abnormal laboratory results upon admission.

Categories of laboratory tests	Test items	Result (reference range)
Complete Blood Count (CBC)	White blood cell count (×10^9^/L)	0.27 (3.5-9.5)
	Neutrophil count(×10^9^/L)	0.06 (1.86-6.3)
	Hemoglobin (g/L)	103 (115-150)
	Platelet count (×10^9^/L)	1 (250-350)
Inflammatory Parameters	PCT (ng/mL)	21.2 (0.00-0.20)
	IL-6 (pg/mL)	1824.6 (0.00-7.00)
	SAA (mg/L)	589.1 (0.00-1.00)
Renal Function	glomerular filtration rate (ml/min)	8 (>80)
	β-2 microglobulin (mg/L)	22.59 (1.0-2.3)
	Urea nitrogen (mmol/L)	38.80 (2.6-7.5)
	Creatinine (umol/L)	481 (49-92)
	Uric acid (umol/L)	827 (155-357)
HLH Diagnostic Markers	Ferritin (ng/mL)	6691.1 (11-308.8)
	sCD25 (U/mL)	3760 (223-710)
	Activated NK Cells %	63.3% (1.9-13.3%)
	Triglyceride(mmol/L)	3.18 (0.4-1.7)
	Fibrinogen (g/L)	6.48 (2-4)
Circulating cytokine levels	IL-6 pg/mL	8294 (<10)
	IL-8 pg/mL	782.2(<15.7)
	IL-10 pg/mL	230.3 (<4.5)
	IFN-γ pg/mL	10.6(<4.4)
	TNF-α pg/mL	34.1(<4.5)
	IL-1β pg/mL	22.5(<2.54)

**Figure 1:**
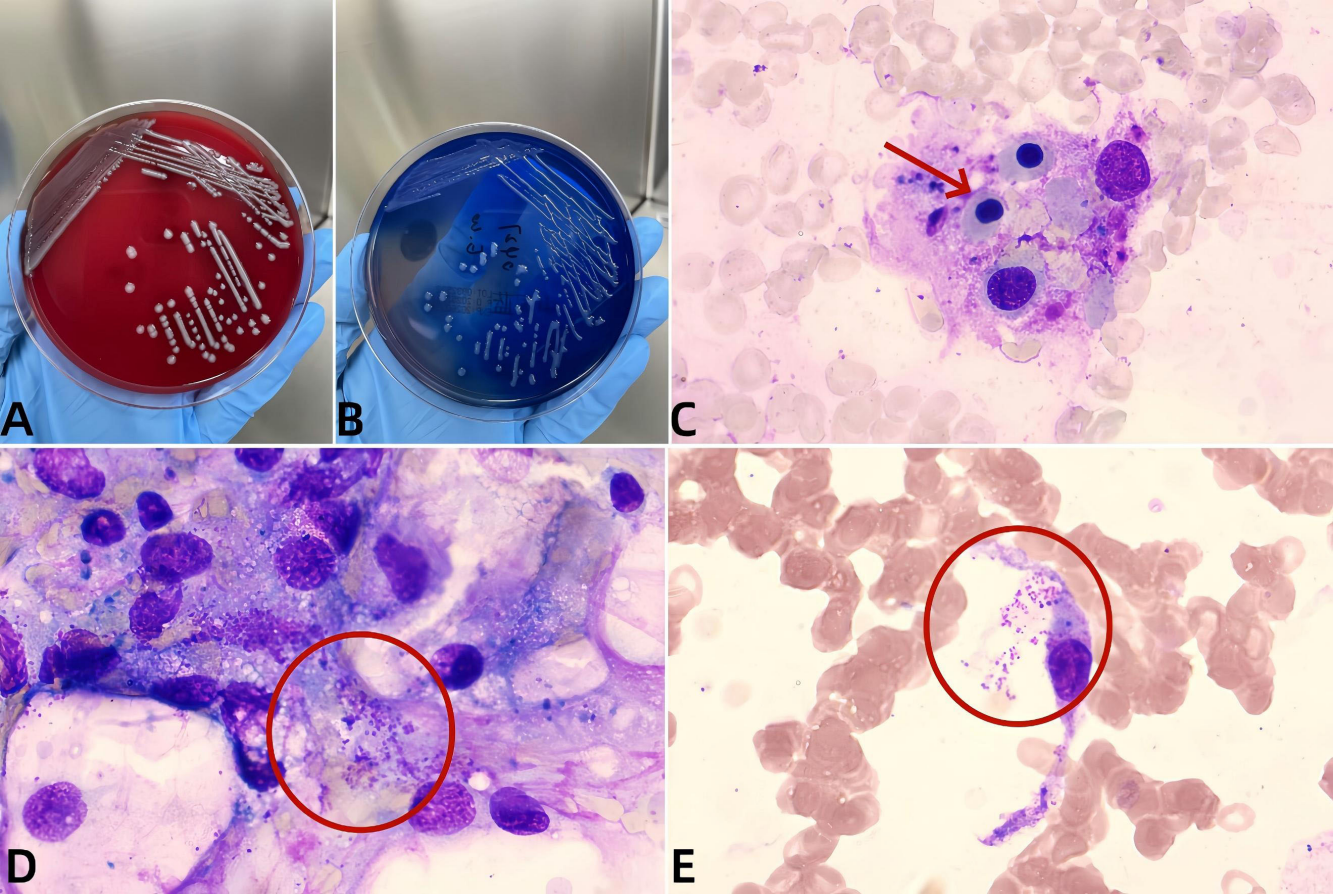
The patient’s blood culture was positive, as indicated by growth on **(A)** Blood agar​ and **(B)** China Blue agar. **(C)** Classical hemophagocytes (engulfing nucleated red blood cells and platelets, indicated by red arrow) were observed in the bone marrow aspirate(Wright-Giemsa stain, 1000×). **(D, E)** Bone marrow smear revealed hemophagocytic cells that had phagocytosed a large number of bacteria, indicated by a red circle (Wright-Giemsa stain, 1000×).

**Figure 2:**
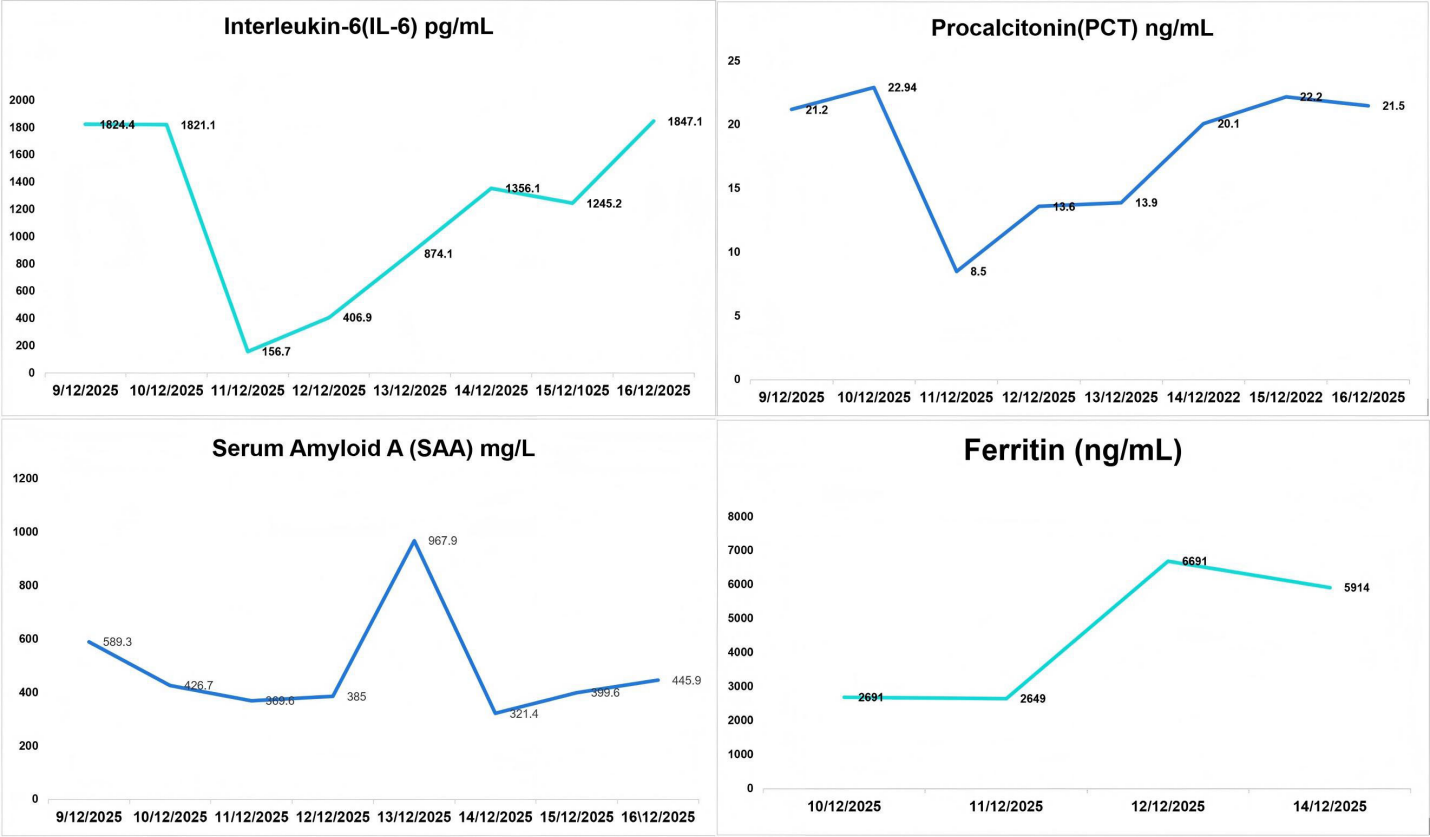
This illustrates the trend of biological markers during the patient’s hospitalization; despite active intervention, no significant improvement was observed.

On the third day of hospitalization, both blood culture and urine culture indicated the presence of Extended-Spectrum Beta-Lactamase-Producing *Escherichia coli* (ESBL-EC) (**Figures 1A, B**). Given the concurrent findings of hyperferritinemia, hypertriglyceridemia, and hypoalbuminemia, HLH secondary to *E. coli* infection was strongly suspected. This suspicion was further supported by the detection of markedly elevated soluble CD25 and circulating cytokine levels. (**Table 1**). Further evaluation with bedside ultrasound revealed mild splenomegaly. Bone marrow aspiration demonstrated 20% active hemophagocytosis, characterized by phagocytosis of numerous bacteria, with no blasts observed. (**Figures 1C–E**). Currently, there is initial evidence pointing toward HLH secondary to *E. coli* infection as a first-line consideration. Also specify which hematologic malignancies are less likely in relation to the presence of blasts(AML, ALL), since lymphomatous bone marrow infiltrations are hematologic malignancies and are not excluded by the absence of blasts. Flow cytometric immunophenotyping of the follow-up bone marrow specimen revealed no abnormal lymphoid or myeloid population, effectively ruling out a hematologic malignancy. Metagenomic pathogen detection identified 48,999 reads of *E. coli*, representing 98% relative abundance, along with 29 reads of the *E. coli* drug-resistance gene *CTX-M-15*-like, with 76% coverage. Based on these results, a diagnosis of HLH secondary to E. coli infection was ultimately confirmed. Based on the antimicrobial susceptibility results(**Table 2**), the treatment regimen was switched to intravenous meropenem at 3g/day for *E. coli* infection. Given the patient’s severe pancytopenia and active infection, dexamethasone (10 mg/m²/day for 3 days) combined with IVIG(0.4 g/kg/day for 3 days) was chosen as initial therapy to avoid the additional myelosuppressive risk associated with etoposide (VP-16). The decision on whether to introduce chemotherapy will be reevaluated once the patient’s clinical status stabilizes. On the sixth day of hospitalization, despite negative blood culture results, various inflammatory markers, including PCT, SAA, and IL-6, and Ferritin, remained persistently elevated (**Figure 2**). The patient succumbed to multiple organ failure on the ninth day of hospitalization. The timeline of disease progression is presented in **Table 3**.

**Table 2:** Antimicrobial susceptibility testing results.

Antimicrobial agent (class)	MIC (mg/L) or zone (mm)	Interpretation (S/I/R)	CLSI breakpoints (S−I−R)
β−LACTAMS/β−LACTAMASE INHIBITOR COMBINATIONS			
Ampicillin/Sulbactam (SAM)	6 mm (KB)	S	≤13−17
Piperacillin/Tazobactam (TZP)	8/4	S	≤8−32
Cefoperazone/Sulbactam (CSO)	16/8	S	≤16−64
Cefotaxime/Sulbactam (CSL)	>0.5/8	R	≤8−64
CARBAPENEMS			
Ertapenem (ETP)	≤0.25	S	≤0.5−2
Imipenem (IPM)	≤1/4	S	≤1−4
Meropenem (MEM)	0.06/1.25	S	≤1−4
CEPHALOSPORINS			
Cefuroxime (CXM)	<16	R	≤4−32
Cefoxitin (FOX)	8	S	≤4−32
Cefpodoxime (CPD)	8	S	≤2−16
Ceftriaxone (CRO)	32	R	≤1−4
Ceftazidime (CAZ)	6	R	≤4−16
MONOBACTAM			
Aztreonam (AZT)	16/8	I	≤8−32
AMINOGLYCOSIDES			
Gentamicin (GEN)	>8	R	≤2−8
Tobramycin (TOB)	>8	R	≤2−8
Amikacin (AMK)	20	S	≤16−20
FLUOROQUINOLONES			
Ciprofloxacin (CIP)	>4	R	≤0.25−1
Levofloxacin (LVX)	>8	R	≤0.5−2
TETRACYCLINES			
Tetracycline (TCY)	>8	R	≤4−16
Minocycline (MIN)	4	S	≤4−16
Tigecycline (TGC)	≤1	S	≤1−4
FOLATE PATHWAY INHIBITORS			
Trimethoprim−Sulfamethoxazole (SXT)	≥4/76	R	≤2−4
PHENICOLS			
Chloramphenicol (CHL)	32	R	≤4−16

**Table 3:** The timeline of disease progression.

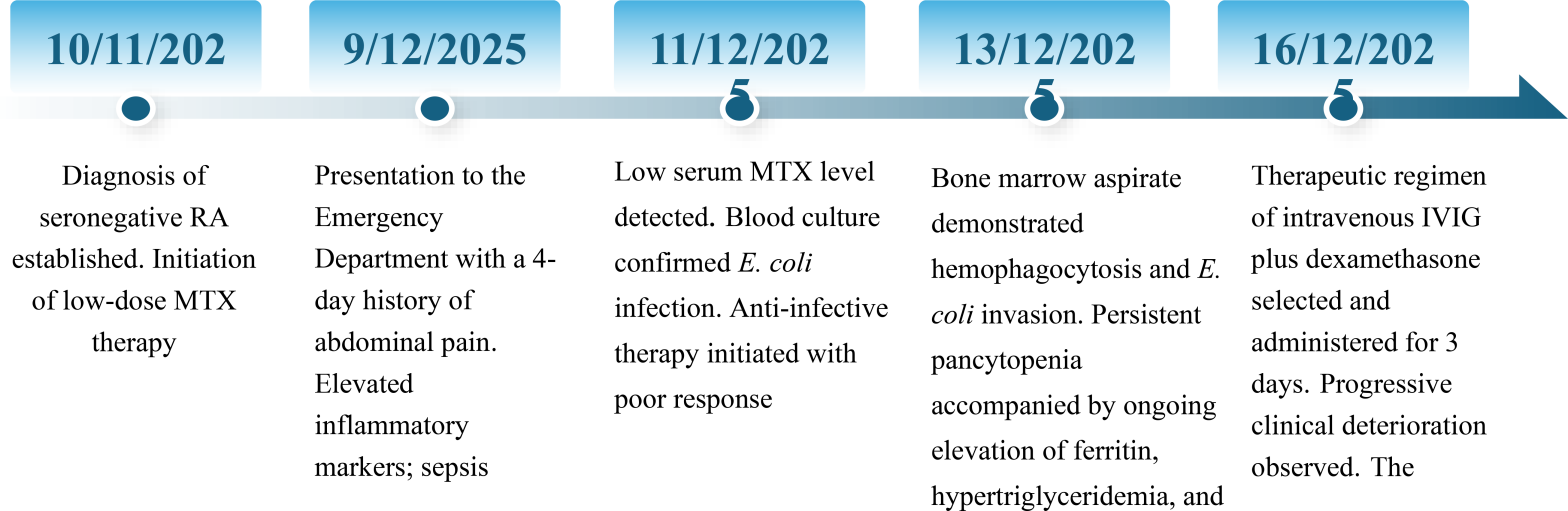				

## 3 Discussion

MTX has remained the standard initial treatment for early RA and has played a role as first-line therapy for most RA patients over the past two decades (6). Higher doses of methotrexate may elevate the risk of cytopenias, infections, and hepatotoxicity. Initially, methotrexate-induced pancytopenia was suspected; however, this was subsequently ruled out by the measurement of a very low plasma methotrexate concentration. Even at low doses, MTX impairs neutrophil chemotaxis in a dose-dependent manner, interfering with immune surveillance and pathogen clearance (7). Low-dose MTX, an immunosuppressive agent, is associated with both an increased risk of infection and greater illness severity in RA patients when infections occur (8). In this clinical context, the isolation of pathogenic bacteria (*E. coli*) in an immunosuppressed patient should be considered high-risk and warrants intervention. One study indicates that approximately 18% of bacteremia cases originate from urinary tract infections with colony counts below 10^5^ CFU/mL (9), underscoring that low-burden infections can also lead to bloodstream infections. The patient’s development of sepsis stemmed from a sequence of events initiated by low-dose MTX-induced immunosuppression: overlooked low-burden urinary colonization, subsequent bacterial translocation into the bloodstream, and a resultant hyperimmune response (cytokine storm), culminating in septic multiple organ dysfunction (cholecystitis, pneumonia, pancytopenia), ultimately triggering HLH. Before initiating immunosuppressive therapy, a comprehensive infectious evaluation must be conducted, and appropriate anti-infective management must be implemented to prevent severe consequences.

A survey revealed that 85% of HLH cases are associated with infectious precipitating​ factors (5). HLH can manifest as either an acute, sepsis-like syndrome or a prodromal phase characterized by mild, gradually evolving symptoms (10). In both presentations, the diagnosis is frequently missed or delayed. Integrating the 2023 HiHASC Adult HLH Consensus (11), the HLH-2004 Criteria (12), and the clinical profile of sepsis, we have established a five-tiered diagnostic framework. The rationale behind this framework is structured as follows: initially ruling out “sepsis mimicking HLH,” subsequently confirming “sepsis-induced HLH,” and ultimately evaluating the need for immunosuppressive therapy. First, clinicians should initiate HLH screening promptly in septic patients when they present with a constellation of findings, including persistent or recurrent unexplained high fever (≥38.5 °C), progressive bi- or tricytopenia, a markedly elevated ferritin level (>3000 μg/L, a strong indicator), or an exponentially increasing trend on monitoring, alongside a poor clinical response to anti-infective and supportive measures. In the second step of rapid differentiation, the following three markers serve as the cornerstone for discrimination between “simple sepsis” and “HLH” in the setting of systemic inflammation: markedly elevated Ferritin​ >10,000 μg/L (specificity >90%), sCD25 exceeding 2,400 U/mL (indicating marked elevation), and concurrently low Fibrinogen​ (<1.5 g/L). Third, failure to observe hemophagocytosis on bone marrow aspiration does not rule out HLH. The fourth step involves a quantitative assessment using the HScore (13), which is calculated from nine parameters: known immunosuppression, fever, organomegaly, cytopenia, ferritin, AST, fibrinogen, triglycerides, and bone marrow hemophagocytosis. A score ≥169 yields a diagnostic specificity of >90% for HLH, whereas a score ≥250 approaches 100% specificity. Compared to the HLH-2004 criteria, the HScore provides a more suitable tool for probabilistic assessment in the context of sepsis. In the setting of sepsis, merely fulfilling 5 criteria according to the HLH-2004 guidelines may lack sufficient diagnostic specificity. Therefore, a comprehensive assessment that incorporates the HScore for probabilistic assessment is advisable. The fifth phase, representing the framework’s cornerstone, emphasizes etiological tracing and therapeutic decision-making. Its objective is to avoid misapplying immunosuppressants to a “septic inflammatory storm,” thereby averting potential harm. If a patient with sepsis continues to deteriorate despite adequate anti-infective therapy and meets the diagnostic criteria of an HScore ≥169 combined with extreme elevation of ferritin and/or sCD25, a diagnosis of sepsis-induced HLH can be confirmed (14). Subsequently, therapeutic management requires a careful balance between infection control and immunosuppression.

In the case of this patient, the clinical diagnosis and treatment were carried out following the steps outlined above. The patient presented to our emergency department with a four-day history of diarrhea and significant pancytopenia. Given a four-week course of immunosuppressive therapy for RA without treatment for an intervening urinary tract infection, along with findings of right upper quadrant tenderness, imaging-confirmed cholecystitis and pneumonia, a positive blood culture for *E. coli*, and markedly elevated inflammatory markers, the initial diagnosis of sepsis was established. Despite symptomatic and supportive care, clinical deterioration persisted. With consent from the family, ferritin and sCD25 were sent for urgent analysis, both returning markedly elevated. Laboratory trends indicate progressive disease, and *E. coli* was also identified in the bone marrow smear. This finding prompted further investigation via pathogenic microorganism sequencing, which confirmed the presence of *E. coli*. The constellation of an HScore of 204 points, fulfillment of 5 HLH-2004 diagnostic criteria, and evidence of infection with *E. coli*, together confirms the diagnosis of infection-associated HLH (**Table 4**). An in-depth literature search across PubMed, EMBASE, and Google Scholar revealed that published case reports (15–17) describing HLH triggered by *E. coli* infection in patients with RA are exceptionally rare. Including the present case, the total number of documented cases is only four (**Table 5**).

**Table 4:** The patient was diagnosed based on the HLH-04 criteria.

The diagnostic criteria for HLH employed in the HLH-2004 trial	The current case
A. The presence of a pathologic mutation in an HLH-associated gene (PRF1, UNC13D, STX11, STXBP2, RAB27A, LYST, SH2D1A, BIRC4, ITK, AP3B1, MAGT1, or CD27)	Not detected
B. Fulfillment of at least five of the eight criteria below establishes the diagnosis.	
1. Fever: Temperature > 38.5 °C.	No
2. Splenomegaly (palpable or radiographically confirmed)	Yes
3. Cytopenias affecting ≥ 2 of 3 lineages in the peripheral blood: hemoglobin < 90 g/L (in infants < 4 weeks: < 100 g/L), platelets < 100 × 10^9^/L, neutrophils < 1.0 × 10^9^/L (Not attributable to bone marrow hypoplasia.).	Yes
4. Hypertriglyceridemia and/or hypofibrinogenemia: Defined as fasting triglycerides > 3.0 mmol/L or fibrinogen < 1.5 g/L.	Yes
5. Hemophagocytosis in bone marrow, spleen, liver, or lymph nodes: Histopathologic evidence of hemophagocytosis without evidence of malignancy.	Yes
6. Low or absent NK-cell activity: Documented by impaired cytotoxic function assay.	No
7. Hyperferritinemia: Serum ferritin ≥ 500 μg/L.	Yes
8. Elevated soluble CD25 (sIL-2R): A recommended cutoff value of ≥ 6400 pg/mL.	Yes
The diagnostic criteria for HLH employed in the HScore.	
1. Known underlying immunosuppression	18 (yes)
2. Temperature (°C)	0
3. Organomegaly	23 (splenomegaly)
4. No. of cytopenias	34 (3 lineages)
5. Ferritin (ng/ml)	50 (6,000)
6. Triglyceride (mmol/liter)	44 (1.5–4)
7. Fibrinogen (gm/liter)	0
8. Serum glutamic oxaloacetic transaminase (IU/liter)	0
9. Hemophagocytosis features on bone marrow aspirate	35 (yes)
10. The total HScore	204

**Table 5:** Literature review: case reports of *E. coli* -associated HLH in patients with RA.

No	Patient profile (RA treatment background)	Infection and HLH triggering event	Outcome	Reference
1	Adult RA patient, long-term use of methotrexate (MTX)​ and sulfasalazine (SSZ)​ with low-dose corticosteroids.	Development of E. coli sepsis.	Resolved​ (condition improved after stopping suspected drugs and controlling infection).	(15)
2	RA patient receiving TNF-α inhibitor (infliximab)​ therapy.	Development of E. coli bacteremia/infection.	Not explicitly stated in the article, but this case is often cited as a cautionary example.	(16)
3	74-year-old female RA patient receiving infliximab​ therapy.	HLH secondary to E. coli pyelonephritis.	Death. Despite aggressive treatment, the patient ultimately could not be saved.	(17)
4	59-year-old female with seronegative RA, treated with methotrexate + prednisone​ for 4 weeks, with an untreated urinary tract infection during this period.	E. coli sepsis​ (positive blood culture), complicated by cholecystitis and pneumonia.	Death. Despite aggressive treatment, the patient ultimately could not be saved.	This case.

Given the patient’s profound cytopenia (platelets 1×10^9^/L, WBC 0.01×10^9^/L) at HLH diagnosis, we opted for a less myelotoxic regimen consisting of corticosteroids and intravenous immunoglobulin (IVIG) as first-line therapy, deferring etoposide to mitigate the risk of life-threatening bone marrow suppression. This approach is supported by current adult HLH guidelines, which emphasize individualized therapy to avoid overtreatment toxicity in high-risk patients (18), and is consistent with real-world evidence demonstrating successful management of secondary HLH with steroid/IVIG-based regimens (19).

## 4 Conclusion

We report a case of HLH secondary to sepsis associated with *E. coli* infection in a seronegative RA patient treated with methotrexate. Methotrexate is not considered a direct cause of HLH but rather a contributing factor to severe infection that leads to HLH, because of its immunosuppressive effects. Unlike previously reported similar cases, in addition to severe bacteremia, bone marrow smear and metagenomic testing identified marrow involvement by *E. coli*, leading to severe immunosuppression. It cannot be excluded that extra-medullary phagocytosis subsequently migrated into the bone marrow in the context of macrophage hyperactivation. This further explains the occurrence of severe pancytopenia and poor prognosis.

## Data Availability

The raw data supporting the conclusions of this article will be made available by the authors, without undue reservation.
