# Supplementary material for: Case Report: Hemophagocytic lymphohistiocytosis secondary to Escherichia coli infiltration of bone marrow in a patient with seronegative rheumatoid arthritis treated with low-dose methotrexate
Source: Front Immunol. 2026 Jul 13;17:1863594. doi: 10.3389/fimmu.2026.1863594 (PMC13402150; doi:10.3389/fimmu.2026.1863594)
Supplement: Supplementary file 1 [file DataSheet1.docx]

| Section | Item # | Description | Reported on Page / Line |
| --- | --- | --- | --- |
| **Title** | 1 | Diagnosis/intervention + “case report” | **Title**: Hemophagocytic lymphohistiocytosis (HLH) secondary to Escherichia coli infiltration of bone marrow in a patient with seronegative rheumatoid arthritis treated with low-dose methotrexate – a case report. **Source: Page 1, Lines 1–3; Page 5, Lines 34–36** |
| **Key Words** | 2 | 2–5 key words (include "case report") | **Keywords**: secondary hemophagocytic lymphohistiocytosis, Escherichia coli, sepsis, bone marrow infiltration, immunosuppression, case report  **Source:** **Page 2, Lines 29–31** |
| **Abstract** | 3a | Uniqueness & scientific contribution | This is the first reported case of extended-spectrum β-lactamase-producing *E. coli* crossing the blood-bone marrow barrier to directly infiltrate bone marrow and trigger fatal secondary HLH in a patient with seronegative RA on low-dose methotrexate (MTX). Only 3 such cases have been published previously; this report expands the evidence base for infection-associated HLH risk stratification in immunosuppressed rheumatic disease populations.  **Source: Page 1, Lines 17–28** |
|  | 3b | Main symptoms & clinical findings | 59-year-old woman with 4-month history of seronegative RA treated with low-dose MTX + prednisone for 4 weeks, with untreated low-burden E. coli bacteriuria. Presented with 4-day abdominal pain, diarrhea, multiple skin ecchymoses, and gingival bleeding. Admission labs showed severe pancytopenia (platelets 1×10⁹/L, WBC 0.27×10⁹/L), multi-organ dysfunction (acute kidney injury, cholecystitis, pneumonia), cytokine storm (ferritin 6691.1 ng/mL, IL-6 8294 pg/mL), and positive blood/urine cultures for E. coli. Bone marrow aspirate revealed 20% hemophagocytosis with intracellular E. coli.  **Source: Page 3, Lines 61–83;** |
|  | 3c | Diagnoses, interventions, outcomes | Final diagnoses: (1) Sepsis secondary to ESBL-producing E. coli with bone marrow infiltration; (2) Secondary HLH; (3) Seronegative RA; (4) Acute kidney injury, acute cholecystitis, pneumonia. Interventions: Empiric meropenem + vancomycin, G-CSF, thrombopoietin, blood transfusion, leucovorin rescue; later switched to high-dose dexamethasone + IVIG for HLH. Outcome: Death from multiple organ failure on hospital day 9.  **Source: Page 4, Lines 85–110** |
|  | 3d | Take-home lesson | Low-burden urinary tract infections in immunosuppressed patients carry a high risk of progression to disseminated sepsis and secondary HLH. Routine pre-immunosuppression infection screening and prompt treatment of even asymptomatic bacteriuria are critical to prevent fatal complications.  **Source: Page 8, Lines 132–134** |
| **Introduction** | 4 | Why this case is unique | HLH is a life-threatening hyperinflammatory syndrome, and sepsis is a well-recognized trigger. However, direct E. coli invasion of the bone marrow niche in this context is exceptionally rare. Low-dose MTX, while first-line for RA, impairs neutrophil chemotaxis and pathogen clearance, creating a permissive environment for occult infection progression. This case addresses a gap in guidance for infection risk mitigation before initiating conventional DMARD therapy.  **Source: Page 2, Lines 35–48;** |
| **Patient Info** | 5a | De-identified demographics | 59-year-old Chinese woman, no other personally identifiable information disclosed. **Source: Page 2, Line 58** |
|  | 5b | Chief complaints | Initial: 4-month recurrent joint swelling, restricted mobility. Later: Frequency, urgency, 4-day progressive abdominal pain, diarrhea, skin ecchymoses, and gingival bleeding. **Source: Page 3, Lines 61–74** |
|  | 5c | History (medical, family, genetic) | No prior history of chronic kidney disease, liver disease, or hematological disorders. No family history of HLH or autoimmune disease. No relevant psychosocial history recorded.  **Source: Page 3, Lines 61–68** |
|  | 5d | Past interventions | Diagnosed with seronegative RA in November 2025; initiated on oral MTX 10 mg weekly + prednisone acetate 15 mg twice daily, no concurrent folic acid supplementation. Urine culture positive for E. coli(colony count <1000 CFU/mL) at RA diagnosis, no antimicrobial therapy given. **Source: Page 3, Lines 65–70** |
| **Clinical Findings** | 6 | Physical exam findings | Soft abdomen with tenderness, rebound tenderness, muscle guarding; positive Murphy’s sign; multiple skin ecchymoses; gingival bleeding; bedside ultrasound confirmed mild splenomegaly and cholelithiasis.  **Source: Page 3, Lines 71–75** |
| **Timeline** | 7 | Timeline of care | **Source: Table 4 (Page 13)** |
| **Diagnostic Assessment** | 8a | Diagnostic testing | Labs: CBC, CRP, PCT, IL-6, SAA, ferritin, sCD25, triglycerides, fibrinogen, cytokine panel, MTX plasma concentration; Pathogen testing: Urine/blood culture, bone marrow smear, mNGS.  **Source: Table 1 (Page 11);** **Page 4, Lines 92–104** |
|  | 8b | Diagnostic challenges | **Source: Page 3, Lines 89–90;** **Page 4, Lines 97–101** |
|  | 8c | Final diagnosis & differentials | Final diagnosis: (1) ESBL-E. colisepsis with bone marrow infiltration; (2) Secondary HLH; (3) Seronegative RA; (4) Acute kidney injury, acute cholecystitis, pneumonia.  Differentials ruled out: MTX-induced pancytopenia (low MTX level), acute leukemia (no blasts on marrow), lymphomatous marrow infiltration (no atypical lymphoid aggregates).  **Source: Page 4, Lines 102–105;** **Table 3 (Page 12)** |
|  | 8d | Prognosis | HScore of 204 (≥169 = >90% specificity for HLH), combined with severe pancytopenia, refractory cytokine storm, and multi-organ failure, indicated near-certain mortality at time of HLH diagnosis.  **Source: Page 6, Lines 153–154; Page 4, Lines 111–114** |
| **Therapeutic Intervention** | 9a | Type of intervention | Antimicrobial therapy, supportive care (hematopoietic growth factors, transfusion, leucovorin rescue), immunomodulatory therapy (corticosteroids, IVIG).  **Source: Page 3, Lines 85–88; Page 4, Lines 106–110** |
|  | 9b | Dosage & duration | Antimicrobial: Meropenem 1.5g/day → escalated to 3g/day after susceptibility results; vancomycin 2g/day. Antimicrobial: Meropenem 1.5g/day → escalated to 3g/day after susceptibility results; vancomycin 2g/day. **Source: Page 3-4, Lines 86, 106;** **Page 4, Lines 107–108** |
|  | 9c | Changes & rationale | Etoposide (VP-16) was deferred due to profound thrombocytopenia (platelets 1×10⁹/L) to avoid life-threatening myelosuppression. First-line therapy was limited to dexamethasone + IVIG per adult HLH guideline recommendations for high-risk patients.  **Source: Page 4, Lines 108–110;** **Page 6-7, Lines 182–188** |
| **Follow-up** and **Outcomes** | 10a | Clinician/patient outcomes | Clinical outcome: Death. The family acknowledged regret regarding the untreated initial bacteriuria and consented to publication to raise clinician awareness. **Source: Page 4, Line 113;** **Page 7, Lines 189–192** |
|  | 10b | Follow-up diagnostics | By hospital day 6, blood cultures were negative, but PCT (21.2 ng/mL), ferritin (6691.1 ng/mL), and IL-6 (8294 pg/mL) remained persistently elevated, indicating unresolved hyperinflammation.  **Source: Page 4, Lines 111–112;** **Table 1 (Page 11)** |
|  | 10c | Adherence & tolerability | All prescribed antimicrobial, supportive, and immunomodulatory therapies were completed as scheduled. No documented drug intolerance or adverse events.  **Source: Page 3- 4, Lines 85–110** |
|  | 10d | Adverse events | No treatment-related adverse events. Death was attributable to disease progression. **Source: Page 5, Line 113** |
| **Discussion** | 11a | Strengths & limitations | Strengths: First documentation of E. coli marrow infiltration triggering HLH, detailed longitudinal diagnostic workflow aligned with 2023 adult HLH consensus. Limitations: Single-case design limits generalizability; no serial marrow sampling to track pathogen clearance kinetics.  **Source: Page 6, Lines 179–181; Page 7, Lines 194–200** |
|  | 11b | Relevant literature | **Table 5**​ (Only 3 cases reported) **Source: Page 13;** **References:15, 16,17** |
|  | 11c | Scientific rationale | Causal chain: Low-dose MTX-induced immunosuppression → untreated low-burden E. coli bacteriuria → bacterial translocation → septic cytokine storm → secondary HLH. Direct detection of E. coli in marrow explains severe pancytopenia and poor response to therapy.  **Source: Page 5, Lines 128–134; Page 7, Lines 194–201** |
|  | 11d | ake-away lesson (no refs) | Clinicians must screen for and treat even asymptomatic, low-colony-count urinary tract infections before initiating MTX therapy in RA patients. For septic patients with ferritin >3000 μg/L and poor response to antimicrobials, prompt HLH evaluation using the HScore is essential to guide balanced infection control and immunomodulatory therapy, avoiding excess toxicity in cytopenic patients.  **Source: Page 5, Lines 132–134; Page 5-6, Lines 139–165** |
| **Patient Perspective** | 12 | Patient/family perspective | Family statement: "We deeply regret not treating the initial low-count E. coli bacteriuria, assuming no symptoms meant no need for intervention. We hope this case reminds clinicians to rule out all infections before starting immunosuppressants, so no other families experience this loss.  **Source: Page 7, Lines 189–192** |
| **Informed Consent** | 13 | Written consent obtained | Written informed consent was obtained from the patient’s legal representative for publication of clinical data and images.  **Source: Page 7, Lines 208–210** |
